# The 26-Item Eating Attitudes Test (EAT-26): Psychometric Properties and Factor Structure in Vegetarians and Vegans

**DOI:** 10.3390/nu15020297

**Published:** 2023-01-06

**Authors:** Courtney P. McLean, Jayashri Kulkarni, Gemma Sharp

**Affiliations:** 1 Department of Neuroscience, Central Clinical School, Monash University, Melbourne, VIC 3800, Australia; 2 Department of Psychiatry, Central Clinical School, Monash University, Melbourne, VIC 3800, Australia

**Keywords:** eating disorder, disordered eating, Eating Attitudes Test, EAT-26, confirmatory factor analysis, veganism, vegetarianism

## Abstract

The eating disorder screener, Eating Attitudes Test (EAT), has been used widely; however, its usability in specific dietary groups such as vegetarians and vegans remain unknown. Considering the rising popularity of vegetarianism and veganism, the current study aimed to assess the psychometric properties and theoretical assumptions of the 26-item EAT in separate groups of vegetarians (*n* = 278), vegans (*n* = 580), and omnivores (*n* = 413). Confirmatory factor analysis of four models from previous literature revealed inadequate fit of the data, with the exception of a 16-item four-factor model in vegetarians and vegans. Further assessment of the original three-factor model and 16-item four-factor model demonstrated poor psychometric properties. The primarily inadequate test–retest reliability discovered in this study, independent of whether a shortened version was used, raises concerns around the utility and stability of the EAT-26 in vegetarians and vegans. Future research should potentially investigate novel ways of measuring eating disorder pathology in these groups.

## 1. Introduction

Eating disorders pose a significant public health issue. With prevalence more than doubling from 3.5% in 2000–2006 to 7.8% in 2012–2018 [1], eating disorders are also associated with high mortality rates and treatment costs [2,3]. Advances to eating disorder prevention, screening, and treatment relies on future research that capitalizes on a public health approach, such as through validating quick, inexpensive, and flexible measures for identifying eating disorder risk [4]. One such self-report measure for the identification of eating disorder pathology in both community and clinical groups is the Eating Attitudes Test (EAT) [5]. The original 40-item EAT was developed in female samples to detect behaviors and attitudes characteristic of anorexia nervosa in the general population [5], with a reduced-item version, the EAT-26, subsequently developed and validated to enable rapid screening of eating disorder pathology [6]. The EAT-26 is rated along a six-point Likert scale to comprise three factors: Dieting, Bulimia and Food Preoccupation, and Oral Control. The EAT-26 has been shown to be accurate in detecting other types of eating disorders, such as bulimia nervosa, beyond the original EAT-40 [7], with a cut-off of 20 or above indicative of disordered eating tendencies. While in recent years, the Eating Disorder Examination Questionnaire (EDE-Q) has been considered the most commonly used self-report measure in community and clinical samples, the EAT is still frequently used by eating disorder researchers, particularly in the US.

The EAT-26 is considered to be psychometrically valid in both community and clinical populations e.g., [8,9], and useful across cultural settings, e.g., [10,11]. However, its use as a case-finding tool is not well supported. For example, items measuring weight-related compensatory behaviors, such as purging, may be of limited use in non-clinical samples with lower prevalence rates [12,13]. Furthermore, a common point of weakness is the tool’s inconsistent factor structure. Conducted in response to suggestions that the EAT-26 may be too long and complex when applied in public health settings, exploratory factor analysis has yielded several alterations to the factor structure of the tool across a range of demographic characteristics, cultures, and languages. A review of published and widely available studies assessing the factor structure of the EAT-26 is presented in Table S1, with the most commonly tested models including:

1. Six-factor model [14]. This 18-item model contains six factors, corresponding to Eating-Related Control, Eating-Related Guilt, Fear of Getting Fat, Food Preoccupation, Social Pressure to Gain Weight, and Vomiting-Purging Behavior.
2. Five-factor model [15]. This model retains 23 items along five factors, corresponding to Fear of Fat, Diet, Other’s Opinions, Preoccupation with Food, and Food Enjoyment.
3. Four-factor model [16]. This 20-item four-factor model retains the original three factors [6], with the addition of a fourth factor, Awareness of Food Contents.
4. Four-factor model [9]. This 16-item four-factor model retains two factors from the original three factors [6], with the addition of Self-Perception of Body Shape and Awareness of Food Contents factors.
5. Unidimensional model [10]. This model sums all of the original 26-items on a one-factor model.

As demonstrated in the literature, there remain large inconsistencies in a commonly accepted alternative factor structure of the EAT-26, raising concerns around the factorial validity of the tool, including its overall structure and utility as a screening measure in community populations.

Vegetarianism, defined as a person who does not eat meat, and veganism, defined as a person who does not eat animal products, has seen increasing popularity over the last decade [17]. This rapid growth is reportedly in response to increasing environmental sustainability concerns as a by-product of animal agriculture, increasing animal welfare concerns, as well as positive benefits to health, such as lower blood pressure, cholesterol, and risk of heart disease compared to omnivore diets [18]. Within the field of clinical eating disorders however, vegetarianism and veganism have long been thought to be related to an increased risk of eating disorder pathology due to the high degree of restriction required to exclude meat and animal products. Conceptualized by Kadambari et al. [19] in the late 1980s, the authors found vegetarian patients with anorexia nervosa were more likely to be dietary abstainers compared to omnivore patients. Since then, the notion that meat-avoidance may provide a socially acceptable way to avoid dietary intake and conceal eating disorder behaviors and attitudes has been widely recognized [20,21,22]. A recent systematic review examining the association between disordered eating and meat-avoidance did not find support for higher rates of disordered eating in these groups [23], and suggested that vegetarians and vegans have been under-researched in the field of eating disorders [23,24]. The review authors comment on the overall poor methodological quality of the studies and suggest that the factorial validity of widely employed eating disorder screening tools may possibly be poor given the low quality of the studies.

Literature to date supporting the validity, reliability, and factor structure of eating disorder scales in vegetarians and vegans is scarce. To our knowledge, no study has examined the factor structure of the EAT in these groups, with much of the limited literature focusing on the EDE-Q [25,26,27]. For example, Heiss et al. [25] were unable to support the factor structure of the original four-factor model and four alternative models of the EDE-Q in self-identified vegans. Further validation by McLean et al. [27] supported inadequate support of these models in a large sample of vegans and omnivores, and extended these findings to vegetarians for the first time. The authors also found overall poor test–retest reliability of the EDE-Q and exploratory factor analysis models, raising concerns around the utility and stability of the tool in these groups. While vegetarian and vegan eating attitudes and behaviors are meaningfully different from omnivores which may erroneously present as increased pathology [22], it is clear that the generalizability of eating disorder tools is implicitly assumed. Taken together, this may ultimately lead to problems with screening and measuring eating pathology in these groups.

As vegetarianism and veganism become more mainstream, it is important that eating disorder tools are robustly validated to accurately capture eating pathology in these groups. In doing so, such findings will support accurate screening, and therefore treatment, of vegetarians and vegans with eating disorders. The aim of the current study was to assess the factor structure and psychometric properties of the EAT-26 in separate samples of vegetarians and vegans, with reference to omnivores. We elected to assess the fit of the original three-factor model and three alternative models in each sample using confirmatory factor analysis. Based on previous literature outlined in Table S1 and more broadly within the vegetarian and vegan eating disorder research field, we expect to find inadequate fit of the data across all tested models. If inadequate fit across all tested models is found, we then aim to identify the tools underlying factor structure using exploratory factor analysis in vegetarians and vegans [28].

## 2. Materials and Methods

Methods for this study received ethics approval by Monash University Human Research Ethics Committee (Project ID: 28501). Prior to participating, all participants were informed of the nature and purpose of the research. The materials and methods for the present study follow that of McLean et al. [27].

### 2.1. Participants

Through social media advertisements, we recruited 1499 participants to the present study. To be eligible, participants must have been residing in Australia and aged 18 years or over. We incorporated Asher et al.’s [29] multi-step process to identifying dietary adherence, whereby participants initially self-identified their diet status (e.g., omnivore, pescatarian, vegan), then selected the animal-based food groups they commonly excluded from their diet on a day-to-day basis (e.g., eggs, poultry, fish and seafood). We then elected to deviate from Asher et al. [29], whereby if misalignment in the multi-step process arose, participants were re-categorized based on the food groups they eliminated [27]. In doing so, we strengthened the validity of each dietary group, resulting in “clean” vegetarian, vegan, and omnivore groups (i.e., vegan participants were ‘vegans’ based on the foods they commonly excluded, rather than self-classification). Due to our focus on vegetarian and vegan diets, we excluded participants who identified as meat-reducers from the final dataset (e.g., flexitarian; *n* = 228), resulting in a total sample of 1271 participants (*M*_age_ = 29.98 years, *SD*_age_ = 9.97 years, 82.00% female, 38.87% Australian, *M*_BMI_ = 24.65, *SD*_BMI_ = 5.21). This can be further broken down into 278 vegetarians (*M*_age_ = 29.37 years, *SD*_age_ = 9.48 years, 87.05% female), 580 vegans (*M*_age_ = 30.34 years, *SD*_age_ = 9.99 years, 83.97% female), and 413 omnivores (*M*_age_ = 29.98 years, *SD*_age_ = 10.27 years, 75.79% female) [27]. A further 223 participants from baseline subsequently participated in a 14-day test–retest study (45 vegetarians, 119 vegans, and 59 omnivores).

### 2.2. Measures

#### 2.2.1. Demographic Information

Participants self-reported a range of demographic characteristics including age, gender, ethnicity, postcode, religion, sexual orientation, highest completed education, and weight and height to calculate their body mass index (BMI; BMI = kg/m^2^). Participants who self-identified as vegetarian, semi-vegetarian, or vegan responded to questions regarding their dietary adherence length (“How long have you followed a vegetarian/vegan diet”), and motivations. Participants were also asked to choose their primary dietary motivation (“What is your primary motivation for eating a vegetarian/vegan diet”) from animal welfare, family tradition, financial, my health, my spiritual beliefs, taste, texture, and/or smell preferences, the environment, weight control, and food insensitivity or intolerance [27].

#### 2.2.2. Eating Attitudes Test (EAT)

The EAT-26 [6] is a self-report measure comprising 26 attitudinal items and five behavioral frequency items designed to identify the presence of eating disorder risk. Participants rate each of the 26 attitudinal items (i.e., EAT-26; e.g., “I give too much time and thought to food”) using a 6-point Likert scale, ranging from *always* to *never*. The EAT-26 comprises three subscales, namely, Dieting, Bulimia and Food Preoccupation, and Oral Control. To score the EAT-26, items 1–25 are re-scored to a 4-point scale, corresponding to *always* (3), *usually* (2), *often* (1), *sometimes* (0), *rarely* (0), and *never* (0), with item 24 reverse-scored. A global or subscale score is calculated by summing all items assigned to the particular scale, with all 26 attitudinal EAT items contributing uniquely to each subscale [6]. The EAT-26 has demonstrated good to excellent internal consistency ranging from 0.86–0.90 [6], with a score of 0.89 for the total sample in the present study.

#### 2.2.3. Eating Disorder Examination-Questionnaire (EDE-Q)

The EDE-Q [30] is a 28-item self-report measure included in the present study to examine the convergent validity of the EAT-26. The EDE-Q assesses core attitudinal and behavioral symptoms of eating disorders over the past 28 days, whereby participants respond to attitudinal items (e.g., “Have you had a definite fear of losing control over eating”) on a 7-point Likert scale, ranging from *not at all* to *markedly*. Comprising of four subscales (Restraint, Eating Concern, Weight Concern, and Shape Concern), the EDE-Q is calculated by totaling the four subscale scores and dividing by the number of subscales (e.g., four). The EDE-Q has been shown to have good to excellent internal consistency ranging from 0.70–0.93 [31], with a score of 0.95 for the total sample in the present study.

#### 2.2.4. Depression, Anxiety, and Stress Scale (DASS-21)

The DASS-21 [32] is a 21-item self-report scale included in the present study to investigate the discriminant validity of the EAT-26. The DASS-21 is designed to measure the negative emotions of depression (e.g., “I found it difficult to work up the initiative to do things”), anxiety (e.g., “I felt I was close to panic”), and stress (e.g., “I felt that I was using a lot of nervous energy”) over the past seven days. The three scales contain seven items rated on a 4-point response scale from *never* to *almost always*. A scale can be computed by totaling the corresponding seven items and multiplying by two, with higher scores indicating greater distress for that particular negative emotional state. The DASS-21 has been shown to have excellent internal consistency ranging from 0.82–0.97 across scales [33], with excellent scores revealed at a total sample level in the present study (0.92, 0.84, and 0.88 for Depression, Anxiety, and Stress scales, respectively).

### 2.3. Procedure

As noted, the procedure for the present study follows that of McLean et al. [27]. Briefly, participants were directed to an online survey and completed demographic characteristic questions, their diet type, and the food groups they commonly exclude on a day-to-day basis. Participants who self-identified as vegetarian, semi-vegetarian, or vegan were then asked questions around the duration and motivation for their dietary adherence. Participants then responded to the EAT-26, EDE-Q, and DASS-21. Upon completion of the online survey, participants completed eating disorder diagnosis and/or mental health diagnosis questions. A subset of participants agreed to take part in future research and were subsequently invited to participate in a 14-day test–retest reliability study. Participants completed identical demographic characteristic and dietary adherence screening questions, and the EAT-26.

### 2.4. Statistical Analysis

Data screening processes, descriptive statistics, reliabilities, and validates were conducting using SPSS Version 27.0 [34], and AMOS Version 26.0 was used to conduct the confirmatory factor analysis [35]. There were six EAT-26 variables with missing data in the vegetarian group, 12 variables in the vegan group, and five variables in the omnivore group, equating to less than 1% missing data in each variable in each group. Missing data was imputed with the variable median, an acceptable approach for low proportions of missing data [36,37]. Sample size recommendations were met for each dietary group using rule of thumb of >200 [38], and the ratio of participants to variables in the EAT-26 (i.e., 10:1) [39].

Confirmatory factor analysis of the EAT-26 was conducted for vegetarian, vegan, and omnivore groups. Analysis was run on separate dietary groups to evaluate model fit for the original three-factor model, unidimensional model, Koslowsky et al.’s [16] 20-item four-factor model, and Ocker et al.’s [9] 16-item four-factor model using the maximum likelihood estimation method [40]. Adequacy of model fit followed Hu and Bentler’s [41] widely accepted two-index presentation strategy, focusing on comparative fit index (CFI) as a relative fit index ≥ 0.95 with a 0.90 cut-off, in combination with the point estimate of root mean square error of approximation (RMSEA) as an absolute index ≤ 0.08. Other fit indices were also reported and considered, including *x*^2^ value, Akaike information criteria (AIC), goodness of fit (GFI), normed fit index (NFI), Tucker–Lewis Index (TLI), and adjusted goodness of fit index (AGFI). Models yielded from previous literature which include factors with less than three items were not evaluated as part of this study (e.g., Maïano et al.’s [14] 18-item six-factor model, Rutt and Coleman et al.’s [15] 23-item five-factor model). This is because models with low numbers of items per factor do not meet minimum recommendations for factor analysis, and therefore were underdetermined [42,43].

Internal consistency of the EAT-26 was calculated using Cronbach’s coefficient alpha (α) in separate dietary groups. Test–retest reliability of the EAT-26 over a 14-day period was estimated using intraclass correlation coefficients (ICC) and 95% confidence interval (CI) on a two-way mixed-effects model with absolute agreement. Pearson correlations I between the EAT-26 and EDE-Q and DASS-21 scales were used to assess convergent and discriminant validity, respectively.

## 3. Results

In the final, overarching sample of the study, 21.87% of participants were vegetarian, 45.63% were vegan, and 32.49% were omnivores. At a total sample level, observed EAT-26 subscale and global scores ranged from 0.00–64.00 for the Global scale, 0.00–35.00 for Dieting, 0.00–16.00 for Bulimia and Food Preoccupation, and 0.00–21.00 for Oral Control. Descriptive statistics for demographic characteristics, including findings of significant differences across dietary groups, have been previously described in McLean et al. [27]. Table 1 presents the EAT-26 scores in vegetarians, vegans, and omnivores. It is of note that Table 1 reports the Chi-Square Test of Independence to determine whether there was a difference in the proportion of participants meeting clinical cut-off proposed by Garner et al. [6] and Orbitello et al. [44] across dietary groups. Using a cut-off score of ≥11, we found a significant association between dietary status and meeting clinical cut-off, with vegetarians (*χ*^2^(1) = 7.44, *p* = 0.006) and vegans (*χ*^2^(1) = 8.75, *p* = 0.003) more likely than omnivores to reach clinical cut-off. Similar findings were found using a cut-off score of ≥20, with vegetarians (*χ*^2^(1) = 5.36, *p* = 0.02) and vegans (*χ*^2^(1) = 16.31, *p* < 0.001) more likely than omnivores to reach clinical cut-off. Proportions did not differ between vegetarians and vegans on either the ≥11 cut-off score (*χ*^2^(1) = 0.06, *p* = 0.81) or ≥20 cut-off score (*χ*^2^(1) = 1.58, *p* = 0.21).

### 3.1. Confirmatory Factor Analysis

We aimed to confirm the factor structure of the original three-factor model and alternate models in separate groups of vegetarians and vegans, with omnivores as a reference group. Confirmatory factor analysis judged model fit to be unacceptable in Garner et al.’s [6] original three-factor model, unidimensional model, and Koslowsky et al.’s [16] 20-item four-factor model across all dietary groups. Based on the two-index presentation strategy cited in the methods, the only model that appeared to have acceptable fit of the data was Ocker et al.’s [9] 16-item four-factor model in vegetarians and vegans, with CFI sitting in the acceptable range (0.90–0.92, respectively) and RMSEA sitting in the borderline acceptable cut-off range (0.09). Table 2 demonstrates the individual model fit indices for each dietary group. Considering the borderline adequate fit of the 16-item four-factor model in vegetarians and vegans, exploratory factor analysis was not conducted [28]. As a result, we assessed the psychometric properties of the original three-factor model and 16-item four-factor model in all subsequent analyses.

### 3.2. Internal Consistency and Test-Retest Reliability

Internal consistency for the EAT-26 ranged from poor (e.g., α = 0.52) to excellent (e.g., α = 0.90) across separate dietary groups (Table 3). Overall, higher internal consistency scores were demonstrated for EAT-26 global scores compared to subscale scores. Scores for the Oral Control subscale sat within the poor to questionable range across dietary groups. Similarly, internal consistency for Ocker et al.’s [9] 16-item four-factor model scores ranged from poor (e.g., α = 0.55) to good (e.g., α = 0.88) across dietary groups. Scores for the Awareness of Food Contents subscale sat within the poor to questionable range.

ICC coefficient values for the EAT-26 sat in the poor range (ICC = 0.11–0.38; Table 3), indicating poor test-retest reliability [45]. For Ocker et al.’s [9] 16-item four-factor model, ICC coefficient values fell in the poor (ICC = 0.21) to moderate (ICC = 0.71) range across dietary populations, indicating poor to moderate test-retest reliability [45].

### 3.3. Convergent and Discriminant Validity

Convergent and discriminant bivariate Pearson correlations were calculated for the EAT-26 with the EDE-Q and DASS-21, respectively (Table 4). Convergent correlations between the EAT-26 and EDE-Q were strong across dietary groups, with correlations between the EAT-26 subscales and EDE-Q ranging from very weak to very strong. Divergent correlations between the EAT-26 and the DASS-21 scales ranged in strength from weak to moderate across dietary groups.

Table 5 demonstrates convergent and discriminant bivariate Pearson correlations between Ocker et al.’s [9] 16-item four-factor model and the EDE-Q and DASS-21, respectively. Convergent correlations between the 16-item four-factor model and EDE-Q ranged in strength from moderate to strong across dietary groups. Divergent correlations between the 16-item four-factor model and the DASS subscales ranged in strength from weak to moderate across dietary groups.

## 4. Discussion

The present study is the first to examine the factor structure and psychometric properties of the EAT-26 in vegetarians and vegans, using omnivores as a reference group. Using a large, robust sample of 278 vegetarians, 580 vegans, and 413 omnivores, we assessed the original three-factor model and alternate models from previous literature using confirmatory factor analysis [9,10,16]. We expected to find inadequate fit of the data in all three dietary groups. Considering the use of the EAT-26 in research settings and for the identification of eating disorder pathology in community and clinical groups, it remains important that the factor structure and psychometric properties of the tool are well-validated in dietary minorities such as vegetarians and vegans. In doing so, we provide advances to the field as the first study to start to validate the EAT-26 in these groups.

In accordance with expectation, we found poor fit of the original three-factor model, unidimensional model, and 20-item four-factor model of the EAT in vegetarians and vegans. This finding is in agreement with previous literature (displayed in Table S1) that the original three-factor model consistently performs poorly across both community and clinical populations, e.g., [9,46,47]. Furthermore, while many studies have examined the theoretical assumptions of the EAT-26, no one study has yet demonstrated the optimal number of subscales or items to achieve ideal specificity and sensitivity in detecting eating disorder pathology in the community [48]. This can be further supported by the higher proportion of vegetarian and vegan participants meeting the clinical cut-off compared to omnivores in the present study. Originally developed in 1982 to screen for anorexia nervosa and other subtypes of eating disorders prior to the popularization of vegetarianism and veganism, it may be possible that the original EAT-26 is not suitable for assessing eating disorder pathology in these groups, as it was never designed to do this.

Despite our findings of inadequate fit of the original three-factor model, confirmatory factor analysis revealed borderline adequate fit of the data using Ocker et al.’s [9] 16-item four-factor model in vegetarians and vegans. This model contains two new factors, Self-Perception of Body Shape, measuring a respondent’s self-esteem related to their body shape, and Awareness of Food Contents, measuring a respondent’s knowledge of the nutritional value of food. Self-Perception of Body Shape also presents as an important extension from Koslowsky et al.’s [16] 20-item four-factor model, which was deemed to be a poor fit for the data in the present study. Comprised of items 1, 11, and 14, which originally sat within the Dieting subscale, this new factor overcomes concerns that some EAT-26 factors contain various distinct theoretical constructs within the one dimension [9]. For example, Ocker et al.’s [9] model supports the idea that perceptions of one’s body image should be an independent construct to dieting [49,50]. Body image satisfaction may be a particularly important concept in vegetarian and vegan populations. Preliminary research suggests that vegans tend to feel more positive and compassionate towards their bodies compared to omnivores, and this is an area that deserves further research exploration in an eating disorder context [22,51,52,53].

Our assessment of the psychometric properties of the EAT-26 demonstrated contrasting results. We discovered overall poor test–retest reliability of the EAT-26, raising concerns around the utility and stability of the tool in all dietary groups. This finding corresponds to previous literature finding poor stability of other eating disorder screening measures such as the EDE-Q in vegetarians and vegans [27]. Although the 16-item four-factor model produced marginally superior test–retest reliability results, it remains difficult to ascertain whether a respondent’s EAT-26 score, independent of whether a reduced-item version of the tool is used, is indicative of their eating pathology severity, or influenced by extraneous factors [54]. Furthermore, we highlight the impact of using a re-categorized four-point Likert scale, meaning that EAT-26 scores offer less variance and provide a lower degree of measurement precision of a respondent’s true eating disorder tendencies [55]. Future research could focus on developing an alternative scoring framework for the EAT-26 guided by Rasch measurement to meet the minimum recommendations of number of categories in a response scale [55], which would in turn increase the reliability and accuracy of the tool [56].

This study has several strengths. First, this study provides the first assessment of the psychometric properties and factor structure of the EAT in vegetarians and vegans. In doing so in a large sample, we were able to investigate vegetarian and vegan groups separately, as literature to date has typically combined meat-avoiders into one group, potentially concealing unique individual group differences [23]. Furthermore, our large sample ensured we reduced the likelihood of overfitting the data, therefore increasing the generalizability and replicability of our results [57]. Next, the findings from this study correspond to emerging literature which suggest that the usability of eating disorder tools in vegetarians and vegans may potentially be inappropriate [25,27]. As previously noted in McLean et al. [27], eating disorder tools were developed prior to the wide acceptance of vegetarianism and veganism (e.g., EAT-26 in 1982, EDE-Q in 1994), and therefore were not co-designed with these dietary minorities in mind, which appear to be here to stay [23]. Considered in the context of literature to date, we recommend the development of a culturally sensitive and novel eating disorder screening tool specific to dietary populations. The proposed screening tool should take into consideration the unique presentation of eating attitudes and behaviors across the meat-avoidance spectrum and incorporate recent changes to eating disorder diagnostic criteria. For example, the tool should incorporate items that disentangle dietary restriction in an attempt to influence weight or shape from cognitive restriction required to ensure meat and/or animal products are not consumed. We also recommend that the proposed tool focus on overcoming some of the limitations of the EAT-26 to incorporate efficient and inexpensive administration and scoring that produces reliable and accurate results. Doing so ensures accuracy in the early identification of vegetarians and vegans who may be at a higher risk of developing eating disorder symptoms.

A limitation of this study is that samples were recruited using convenience sampling, particularly special interest dietary groups on social media, and therefore generalizability of the findings may be limited to broader populations. However, the large sample size of vegetarians, vegans, and omnivores and the collection of important sociodemographic factors in this study did allow for a comprehensive suite of psychometric properties to be conducted on the EAT-26. Second, there was a low response rate of participants taking part in the test–retest reliability arm of the study, which remains a critical limitation across retest research [58]. With little known about vegetarian and vegan clinical samples and the eating behaviors of meat-reducers (i.e., semi-vegetarians, flexitarians) [59], future research is needed to explore the unique presentation of eating pathology in these groups, if any, and how this presentation may differ from omnivores. This would ultimately provide clinicians with information around how to best support vegetarians and vegans seeking eating disorder treatment. Lastly, in order to provide a holistic viewpoint to the relationship between vegetarianism, veganism, and eating disorder pathology, future research could also examine orthorexia nervosa. Though not formally categorized in the Diagnostic and Statistical Manual of Mental Disorders (DSM-5) [60], orthorexia nervosa is defined as an unhealthy fixation on eating “healthy” foods and has been characterized as having numerous commonalities with vegetarian and vegan diets (e.g., nutritional rules becoming an increasingly significant part of one’s day) [61,62]. By incorporating orthorexia nervosa tools into future assessments of eating pathology in vegetarian and vegan research, we can begin to understand how these constructs may interrelate.

This study has notable implications for clinical practice. First, we highlight the importance of understanding vegetarian and vegan clients’ dietary motivations and potential maintenance factors. Understanding how these factors fit into an individual’s eating disorder and mental health history will allow for a more informed and contextual understanding of their eating disorder results (e.g., on the EAT-26), further ensuring that they are not being over-pathologized for simply following their chosen diet. As previously noted, we highlight that higher eating pathology scores could be driven by simple adherence to a vegetarian and vegan diet, but, nevertheless, could be related to the masking of potential eating pathology [27]. Thoroughly assessing an individual’s motivations for dietary adherence at the commencement and throughout treatment will allow the person and their clinical team to continuously re-evaluate how their dietary choices are impacting their recovery. Overall, we encourage a comprehensive assessment and individualistic case-by-case approach rather than solely focusing on measure scores to evaluate eating disorder pathology [27].

In conclusion, this study examined the factor structure and psychometric properties of the EAT-26 in vegetarians and vegans for the first time. We found borderline adequate fit of a 16-item four-factor model in vegetarians and vegans, suggesting that this model is better suited at quantifying eating pathology in these groups compared to other tested models. Overall, our finding of poor test–retest reliability of the EAT-26 in vegetarians and vegans raises concerns around the usability and accuracy of the tool when applied to community and clinical populations. Given the present study’s findings, we cannot be certain that the higher proportion of vegetarian and vegan participants meeting clinical cut-off compared to omnivores is accurate. While the 16-item four-factor model produced marginally better psychometric properties than the full length EAT-26, we encourage caution when interpreting results in vegetarians and vegans until future research considers new novel ways of measuring eating disorders in these groups. Doing so will ensure accuracy in capturing unique vegetarian and vegan eating attitudes and behaviors.

## Figures and Tables

**Table 1:** Eating Attitudes Test (EAT) scores in vegetarians, vegans, and omnivores.

	Total (*n* = 1271)	Vegetarian (*n* = 278)	Vegan (*n* = 580)	Omnivore (*n* = 413)	Statistics
Global	9.54 (10.52)	9.80 (10.72)	10.47 (11.34)	8.07 (8.92)	*F*(2,1268) = 6.41, *p* = 0.002, η^2^ = 0.01 *
Clinical cut-off					
≥20% (*n*) ^1^	13.77 (175)	14.03 (39)	17.42 (101)	8.48 (35)	*χ*^2^(2) = 16.26, *p* < 0.001 **
≥11% (*n*) ^2^	28.95 (368)	32.37 (90)	31.55 (183)	23.00 (95)	*χ*^2^(2) = 10.60, *p* = 0.005 *
Dieting	6.18 (7.01)	6.36 (7.23)	6.75 (7.44)	5.24 (6.09)	*F*(2,1268) = 5.75, *p* = 0.003, η^2^ = 0.009 *
Bulimia	1.55 (2.92)	1.55 (2.95)	1.83 (3.13)	1.18 (2.52)	*F*(2,1268) = 6.02, *p* = 0.002, η^2^ = 0.009 *
Oral Control	1.81 (2.45)	1.89 (2.62)	1.89 (2.57)	1.65 (2.14)	*F*(2,1268) = 1.31, *p* = 0.270, η^2^ = 0.002

Note. *M*(*SD*) reported for ordinal variables. ^1^ Cut-off score proposed by Garner et al. [6]. ^2^ Cut-off score proposed by Orbitello et al. [44]. Between-subjects one-way ANOVA with main effect of dietary group (e.g., vegetarian, vegan, omnivore) were used to evaluate dependent variables; η^2^ = eta-squared; Chi-Square Test of Independence were used to evaluate the distribution of dietary group on clinical cut-off scores; statistics column relates to omnibus tests (all levels for categorical row variables); values for EAT-26 were generated using imputed datasets. Bulimia = Bulimia and Food Preoccupation subscale. * *p* = 0.01, ** *p* < 0.001.

**Table 2:** Confirmatory factor analysis fit indices of the original and alternative models of the Eating Attitudes Test (EAT) in vegetarians, vegans, and omnivores.

	*x*^2^ (*df*)	AIC	CFI	RMSEA	GFI	NFI	TLI	AGFI
Three-factor model								
Vegetarian	1273.28 (296) *	1383.28	0.72	0.11 [0.10,0.12]	0.76	0.67	0.70	0.72
Vegan	1651.93 (296) *	1761.93	0.80	0.09 [0.09,0.09]	0.81	0.77	0.78	0.77
Omnivore	1692.26 (296) *	1802.26	0.68	0.11 [0.10,0.11]	0.75	0.64	0.65	0.70
Unidimensional model								
Vegetarian	1710.01 (299) *	1814.01	0.60	0.13 [0.13,0.14]	0.68	0.56	0.57	0.62
Vegan	2227.51 (299) *	2331.51	0.71	0.11 [0.10,0.11]	0.74	0.68	0.69	0.70
Omnivore	1865.42 (299) *	1969.42	0.64	0.11 [0.11,0.12]	0.72	0.60	0.61	0.67
20-item four-factor model ^a^								
Vegetarian	508.66 (164) *	600.66	0.87	0.09 [0.08,0.10]	0.84	0.82	0.85	0.80
Vegan	904.77 (164) *	996.77	0.87	0.09 [0.08,0.09]	0.85	0.84	0.84	0.81
Omnivore	722.00 (164) *	854.00	0.83	0.09 [0.08,0.10]	0.85	0.79	0.80	0.81
16-item four factor model ^b^								
Vegetarian	325.90 (98) *	433.90	0.90	0.09 [0.09,0.10]	0.88	0.87	0.88	0.83
Vegan	526.17 (98) *	634.17	0.92	0.09 [0.08,0.09]	0.90	0.90	0.90	0.86
Omnivore	463.42 (98) *	571.42	0.88	0.09 [0.09,0.10]	0.89	0.86	0.86	0.64

Note. AIC = Akaike information criteria, CFI = comparative fit index, RMSEA = root mean square error of approximation, GFI = goodness of fit index, NFI = formed fit index, TLI = Tucker–Lewis index, AGFI = adjusted goodness of fit index. * *p* < 0.001. *n* = 1271 (278 vegetarian, 580 vegan, 413 omnivore). ^a^ 20-item four-factor model = Dieting (item 1, 10, 11, 12, 14, 22, 23, 24), Oral Control (item 8, 13, 26), Awareness of Food Contents (item 6, 7, 16, 17), Food Preoccupation (item 3, 4, 18, 19, 21). ^b^ 16-item four-factor model = Self-perception of Body Shape (item 1, 11, 14), Dieting (item 10, 12, 22, 23, 24), Awareness of Food Contents (item 6, 7, 16, 17), Food Preoccupation (item 3, 4, 18, 21).

**Table 3:** Internal consistency and test-retest reliability of the Eating Attitudes Test (EAT) in vegetarians, vegans, and omnivores.

		Internal Consistency (α)		
		Vegetarian	Vegan	Omnivore
EAT-26	Global	0.89	0.90	0.86
	Dieting	0.86	0.86	0.81
	Bulimia	0.80	0.79	0.80
	Oral Control	0.65	0.65	0.52
Four-factor model ^a^	Body Shape	0.88	0.88	0.85
	Dieting	0.80	0.81	0.69
	Food Contents	0.55	0.62	0.64
	Food Preoccupation	0.85	0.85	0.84
		Test-retest Reliability (ICC [95% *CI*])		
		Vegetarian	Vegan	Omnivore
EAT-26	Global	0.21 [0.18,0.25]	0.22 [0.19,0.25]	0.17 [0.15,0.20]
	Dieting	0.29 [0.24,0.34]	0.28 [0.25,0.32]	0.23 [0.190.,26]
	Bulimia	0.36 [0.30,0.43]	0.35 [0.29,0.41]	0.38 [0.32,0.43]
	Oral Control	0.19 [0.14,0.24]	0.18 [0.14,0.22]	0.11 [0.08,0.15]
Four-factor model ^a^	Body Shape	0.71 [0.65,0.76]	0.70 [0.67,0.74]	0.65 [0.61,0.70]
	Dieting	0.43 [0.37,0.50]	0.45 [0.40,0.49]	0.29 [0.25,0.35]
	Food Contents	0.21 [0.15,0.28]	0.26 [0.21,0.32]	0.29 [0.23,0.34]
	Food Preoccupation	0.57 [0.50,0.63]	0.56 [0.50,0.61]	0.55 [0.49,0.60]

Note. ^a^ Ocker et al.’s [9] 16-item four-factor model, Bulimia = Bulimia and Food Preoccupation, Body Shape = Self-perception of Body Shape, Food Contents = Awareness of Food Contents, ICC = intraclass correlation coefficient, *CI* = confidence interval. *n* = 1271 (278 vegetarian, 580 vegan, 413 omnivore), test–retest *n* = 223 (45 vegetarian, 119 vegan, 59 omnivore).

**Table 4:** Convergent and discriminant validity of the Eating Attitudes Test in vegetarians, vegans, and omnivores.

	1	2	3	4	5	6	7	8
Vegetarian								
1. EAT Global	-							
2. EAT Dieting	0.95 **	-						
3. EAT Bulimia	0.81 **	0.69 **	-					
4. EAT Oral Control	0.56 **	0.36 **	0.27 **	-				
5. EDE-Q	0.77 **	0.80 **	0.64 **	0.19 **	-			
6. DASS Depression	0.37 **	0.35 **	0.36 **	0.15 *	0.49 **	-		
7. DASS Anxiety	0.41 **	0.36 **	0.36 **	0.26 **	0.40 **	0.64 **	-	
8. DASS Stress	0.37 **	0.36 **	0.36 **	0.12*	0.43 **	0.70 **	0.73 **	-
Vegan								
1. EAT Global	-							
2. EAT Dieting	0.96 **	-						
3. EAT Bulimia	0.83 **	0.73 **	-					
4. EAT Oral Control	0.62 **	0.46 **	0.32 **	-				
5. EDE-Q	0.79 **	0.80 **	0.71 **	0.31 **	-			
6. DASS Depression	0.39 **	0.38 **	0.36 **	0.21 **	0.46 **	-		
7. DASS Anxiety	0.38 **	0.34 **	0.36 **	0.27 **	0.42 **	0.69 **	-	
8. DASS Stress	0.42 **	0.39 **	0.40 **	0.22 **	0.47 **	0.69 **	0.76 **	-
Omnivore								
1. EAT Global	-							
2. EAT Dieting	0.95 **	-						
3. EAT Bulimia	0.82 **	0.69 **	-					
4. EAT Oral Control	0.51 **	0.28 **	0.27 **	-				
5. EDE-Q	0.71 **	0.74 **	0.64 **	0.01	-			
6. DASS Depression	0.36 **	0.33 **	0.33 *	0.19 **	0.46	-		
7. DASS Anxiety	0.35 **	0.27 **	0.33 *	0.29 **	0.65 **	0.65 **	-	
8. DASS Stress	0.37 **	0.31 **	0.36 **	0.22 **	0.46 **	0.69 **	0.73 **	-

Note. Bulimia = Bulimia and Food Preoccupation subscale, ** *p* < 0.01, * *p* < 0.05. *n* = 1271 (278 vegetarian, 580 vegan, 413 omnivore).

**Table 5:** Convergent and discriminant validity of Ocker et al.’s [9] 16-item four-factor model of the Eating Attitudes Test in vegetarians, vegans, and omnivores.

	1	2	3	4	5	6	7	8
Vegetarian								
1. EAT Body Shape	-							
2. EAT Dieting	0.78 **	-						
3. EAT Food Contents	0.46 **	0.61 **	-					
4. EAT Food Preoccupation	0.67 **	0.65 **	0.48 **	-				
5. EDE-Q	0.80 **	0.75 **	0.51 **	0.64 **	-			
6. DASS Depression	0.43 **	0.30 **	0.08	0.36 **	0.49 **	-		
7. DASS Anxiety	0.38 **	0.34 **	0.12 *	0.35 **	0.40 **	0.64 **	-	
8. DASS Stress	0.41 **	0.32 **	0.15 *	0.34 **	0.43 **	0.70 **	0.73 **	-
Vegan								
1. EAT Body Shape	-							
2. EAT Dieting	0.75 **	-						
3. EAT Food Contents	0.45 **	0.62 **	-					
4. EAT Food Preoccupation	0.73 **	0.69 **	0.45 **	-				
5. EDE-Q	0.82 **	0.74 **	0.49 **	0.70 **	-			
6. DASS Depression	0.44 **	0.35 **	0.13 **	0.35 **	0.46 **	-		
7. DASS Anxiety	0.39 **	0.35 **	0.11 **	0.35 **	0.42 **	0.69 **	-	
8. DASS Stress	0.45 **	0.36 **	0.17 **	0.40 **	0.47 **	0.69 **	0.76 **	-
Omnivore								
1. EAT Body Shape	-							
2. EAT Dieting	0.70 **	-						
3. EAT Food Contents	0.40 **	0.67 **	-					
4. EAT Food Preoccupation	0.65 **	0.66 **	0.48 **	-				
5. EDE-Q	0.76 **	0.66 **	0.43 **	0.65 **	-			
6. DASS Depression	0.36 **	0.29 **	0.13 **	0.34 **	0.46 **	-		
7. DASS Anxiety	0.34 **	0.24 **	0.09	0.34 **	0.39 **	0.65 **	-	
8. DASS Stress	0.35 **	0.28 **	0.11 *	0.38 **	0.46 **	0.69 **	0.73 **	-

Note. Body Shape = Self-perception of Body Shape subscale, Food Contents = Awareness of Food Contents subscale. ** *p* < 0.01, * *p* < 0.05. *n* = 1271 (278 vegetarian, 580 vegan, 413 omnivore).

## Data Availability

Data from this study cannot be made available in line with ethics compliance.

## Supplementary Materials

The following supporting information can be downloaded at: https://www.mdpi.com/article/10.3390/nu15020297/s1, Table S1: Literature review of studies examining the factor structure of EAT-26 and EAT-26 adult adaptations. References [6,8,9,10,11,14,15,16,46,47,63,64,65,66,67,68,69,70,71,72,73,74,75,76,77,78,79,80,81,82] are cited in the supplementary materials.
