# Supplementary material for: The 26-Item Eating Attitudes Test (EAT-26): Psychometric Properties and Factor Structure in Vegetarians and Vegans
Source: Nutrients. 2023 Jan 6;15(2):297. doi: 10.3390/nu15020297 (PMC9864971; doi:10.3390/nu15020297)
Supplement: Supplementary file 1 [file nutrients-15-00297-s001.zip › nutrients-2143252-supplementary.pdf]

Table S1. Literature review of studies examining the factor structure of the EAT-26 and EAT-26 adult adaptations

Author (Year)	EAT Version	Country/ Language	Study Population	*N*	Summary of Factor Analysis
Ahmadi et al. [1]	EAT-26	Iran/Persian	Female university students	598	- CFA of the three- and five-factor model indicated poor fit.<br>- EFA indicated five factors with 24 items corresponding to Drive for Thinness (item 1, 10, 11, 12, 14, 22, 23, 24), Restrained Eating (item 2, 5, 6, 7, 16, 17), Perceived Social Pressure to Eating (item 8, 13, 20), Food Preoccupation and Oral Control (item 15, 18, 19, 21), and Bulimia (item 3, 4, 9, 10). Items 25 and 26 were discarded.
Ambrosi-Randić and Pokrajac-Bulian [2]	EAT-26	Croatia/Croatian	Females aged 10-25 years	1,396	- EFA of the high school sample indicated three factors 26 items corresponding to Bulimia and Food Preoccupation (item 1, 3, 4, 6, 10, 11, 12, 14, 18, 19, 21, 22), Dieting (item 2, 5, 7, 16, 17, 23, 24, 25), and Social Pressure (8, 9, 13, 15, 20, 26). EFA of the university sample indicated three factors with 26 items corresponding to Bulimia and Food Preoccupation (item 3, 4, 10, 14, 18, 19, 21, 26), Dieting (item 1, 2, 6, 7, 11, 12, 16, 17, 22, 23, 24, 25), and Social Pressure (item 5, 8, 9, 13, 15, 20).
Belon et al. [3]	EAT-26	United States/ English	Caucasian and Hispanic female university students	235	- CFA of the original three-factor model and Rutt and Coleman’s [4] 17-item five-factor model indicated poor fit. Ocker et al.’s [5] 16-item four-factor model indicated acceptable fit in both Caucasian and Hispanic groups.
Choudry and Mumford [6]	EAT-26	Pakistan/Urdu	Female children aged 12-16 years.	271	- EFA indicated four factors with 20 items corresponding to Dieting (item 1, 6, 7, 11, 12, 17, 19, 23), Discomfort (item 9, 10, 15, 21, 22, 24), Food Preoccupation (item 2, 3, 25), Social Pressure (item 8, 13, 20, 25). Items 4, 5, 14, 16, 18, 26 were discarded.
Doninger et al. [7]	EAT-26	United States/ English	Female university athletes	207	- CFA of the original three-, Koslowsky et al.’s [8] four-, and Rutt and Coleman’s [4] five-factor model indicated poor fit.<br>- EFA indicated five factors with 20 items corresponding to Drive for Thinness (item 1, 2, 10, 11, 12, 14, 23), Food Preoccupation (item 3, 18, 21), Others’ Perceptions (item 8, 13, 20), Purging Behaviour (item 9, 26), Dieting Behavior (item 6, 7, 16, 17, 23). Items 4, 5, 15, 19, 22, 25 were discarded.
Dotti and Lazzari [9]	EAT-26	Italy/Italian	High school students	1,227	- EFA yielded a three-factor model, corresponding to Dieting, Bulimia and Food Preoccupation, and Oral Control, and five-factor model, with the addition of Awareness of Food Content and Vomiting factors.
Douka et al. [10]	EAT-26	Greece/Greek	University students and female ED patients	187	- EFA indicated a three-factor model with 13 items corresponding to Food Preoccupation (item 10, 11, 12, 14, 18, 22), Dieting (item 6, 16, 17, 19), Important Others (item 8, 13, 20). Items 1,2 3, 4, 5, 7, 9, 15, 21, 23, 24, 25, 26 were discarded.
Engelsen and Hagtvet [11]	EAT-12	Norway/English	Children aged 14-15 years	485	- CFA of the 12-item three- and one-factor models indicated poor fit. An 11-item three-factor model (discarding item 11) in girls and a 9-item three-factor model (discarding items 2, 5, 11) in boys provided the best fit of the data.
Fekih-Romdhane et al. [12]	EAT-7	Lebanon/Arabic	Community sample	1,175	- EFA revealed a 10-item one-factor solution (item 2, 3, 4, 6, 7, 15, 16, 17, 22, 23).<br>- CFA of the EFA model provided poor fit. A reduced 7-item model provided excellent fit of the data (item 2, 6, 7, 15, 16, 17, 22).
Haddad et al. [13]	EAT-26	Lebanon/Arabic	Community sample	811	- EFA indicated six factors with 26 items corresponding to Restrained Eating Attitude (item 5, 6, 7, 16, 17, 19), Anorexic Attitude (item 1, 2, 10, 11, 12, 14), Cycle of Bulimic Crises (representing two factors, item 3, 4, 8, 18, 21, 22, 23, 24, 25), Impact of Others Perception (item 8, 13, 20), Choice of Food Quality (item 15, 26).<br>- CFA of the EFA model indicated adequate fit.

Johnson and Bedford [14]	EAT-26	Canada/English	Community sample	343	- EFA indicated six factors with 21 items corresponding to Food Preoccupation (item 3, 6, 10, 12, 18, 21, 22), Dieting (item 7, 16, 17), Image Preoccupation (item 1, 11, 14), External Perception (8, 13, 20), Oral Control (5, 15), Self Control (item 4, 19, 25). Items 2, 9, 19, 23, 24, 26 were discarded.
Kelly et al. [15]	EAT-26	United States/ English	Female university students	2,208	- CFA of the EAT-26 did not support invariance of factor loadings and invariance of intercepts across White and Black samples.
Khaled et al. [16]	EAT-26	Qatar/Arabic	Female university students	2,692	- EFA indicated five factors with 19 items corresponding to Fear of Getting Fat, Eating-related Control, Food Preoccupation, Social Pressure to Gain Weight, and Vomiting-purging Behaviour. Items 2, 5, 15, 18, 22, 24, 26 were discarded.
King and Bhugra [17]	EAT-26	India/Hindi	Females aged 14-23	574	- EFA yielded 10 factors with no discernible pattern of items.
Koslowsky et al. [8]	EAT-26	Israel/Hebrew	Female soldiers	809	- EFA indicated four factors with 20 items corresponding to Dieting (item 1, 10, 11, 12, 14, 22, 23, 24), Oral Control (item 8, 13, 26), Awareness of Food Contents (item 6, 7, 16, 17), Food Preoccupation (item 3, 4, 18, 19, 21). Items 2, 5, 9, 15, 20, 25 were discarded.
Lavik et al. [18]	EAT-12	Norway/English	Children aged 13-18 years.	2,024	- EFA indicated three factors with 11 items corresponding to Dieting (item 1, 2, 3, 4), Bulimia and Food Preoccupation (item 6, 7, 8), Oral Control (item 9, 10, 11, 12). Item 5 was discarded.
Lee and Lee [19]	EAT-26	Hong Kong/ Chinese	Female children aged 15-18 from Hong Kong, Shenzhen, and Hunan	796	- EFA indicated a 24-item three-factor model in the Hong Kong sample corresponding to Fat Concern and Dieting (item 1, 2, 6, 7, 11, 12, 14, 16, 17, 22, 23, 24), Food Preoccupation (item 3, 4, 10, 18, 21), Social Pressure to Eat (item 5, 8, 9, 13, 15, 20, 26). EFA indicated a 20-item three-factor model in the Shenzhen sample corresponding to Fat Concern and Dieting (item 1, 2, 6, 7, 11, 12, 14, 16, 17, 22, 23), Food Preoccupation (item 3, 4, 10, 21), Social Pressure to Eat (item 6, 13, 15, 18, 20). EFA indicated a 19-item three-factor model in the Hunan sample corresponding to Fat Concern and Dieting (item 1, 2, 6, 11, 12, 14, 16, 17, 22), Social Pressure to Eat (item 5, 7, 8, 13, 15, 19, 20), Vomiting (item 9, 10, 26).
Maïano et al. [20]	EAT-26	France/French	Children aged 11-18 years.	1,035	- CFA of six models with three to eight factors were examined, with the 26-item six-factor model providing the best fit of the data.<br>- EFA of the 26-item six factor model indicated eight items were problematic (items 2, 3, 4, 15, 19, 23, 24, 25), resulting in an 18-item six-factor model.<br>- CFA in a new sample confirmed acceptable fit of the 18-item six-factor model, with factors corresponding to Eating-related Control (item 5, 6, 7, 16, 17), Eating-related Guilt (item 10, 22), Fear of Getting Fat (item 1, 11, 12, 14), Food Preoccupation (item 18, 21), Social Pressure to Gain Weight (item 8, 13, 20), Vomiting-purging Behaviour (item 9, 26).
McEnery et al. [21]	EAT-26	Ireland/English	Children aged 12-18 years.	2,444	- CFA of the original 26-item three-factor model and 26-item unidimensional model indicated poor fit of the data. Maïano et al.'s [20] 18-item six-factor model indicated excellent fit of the data.
Mumford et al. [22]	EAT-26	Pakistan/English	Children aged 14-16 years	369	- EFA revealed four factors with 26 items corresponding to Dieting (item 1, 2, 6, 7, 10, 11, 12, 14, 16, 17, 19, 22, 23, 24), Food Preoccupation (item 3, 4, 10, 18, 21), Social Pressure (item 5, 8, 11, 13, 20), Vomiting (item 2, 9, 26). Item 2 loaded onto both Dieting and Vomiting subscales.
Mumford et al. [23]	EAT-26	England/English	Female children aged 14-16 years	559	- EFA supported a 26-item four-factor solution in an Asian sample corresponding to Dieting (item 1, 2, 6, 7, 10, 11, 12, 14, 16, 17, 22, 23, 24), Social Pressure (item 5, 8, 13, 15, 20), Food Preoccupation (item 3, 4, 18, 21, 25), Vomiting (item 9, 19, 26). EFA supported a 24-item four-factor solution in a Caucasian sample

					corresponding to Dieting (item 1, 2, 6, 7, 10, 11, 12, 14, 16, 17, 22, 23, 24), Social Pressure (item 8, 13, 20), Food Preoccupation (item 3, 4, 18, 21, 25), Vomiting (item 9, 24, 26).
Ocker et al. [5]	EAT-26	United States/ English	Female university students	785	- CFA of Garner et al.'s [24] 26-item three-factor model and Koslowsky et al.'s [8] 20-item four-factor model indicated poor fit of the data.<br>- EFA indicated four factors with 16 items corresponding to Self-perception of Body Shape (item 1, 11, 14), Dieting (item 10, 12, 22, 23, 24), Awareness of Food Contents (item 6, 7, 16, 17), Food Preoccupation (item 3, 4, 18, 21). Items 2, 5, 8, 9, 13, 15, 19, 20, 25, 26 were discarded.
Park and Beaudet [25]	EAT-26	Canada/English	Females with weight concerns.	3,792	- EFA yielded four factors with 21 items corresponding to Food Preoccupation (item 3, 4, 10, 18, 21, 22), Body Image Preoccupation (item 1, 11, 12, 14), Self-imposed Dieting (item 6, 7, 16, 17, 19, 23), Perceived External Pressure to Eat (item 8, 13, 20). Items 2, 5, 15, 24, 26 were discarded.
Rivas et al. [26]	EAT-26	Spain/Spanish	Females aged 12-21 years with and without an ED	1,148	- EFA supported a 26-item unidimensional model in separate non-clinical and clinical samples.
Rogoza et al. [27]	EAT-26	Poland/English	Female children and university students	617	- CFA confirmed a 13-item bi-factor model with four factors corresponding to Social Pressure (item 8, 13, 20), Food Awareness (item 6, 7, 16, 17), Bulimia (item 9, 25), Food Preoccupation (item 3, 4, 18, 21).
Rutt and Coleman [4]	EAT-26	United States/ English	Hispanic university students	255	- CFA of the original three-factor model indicated poor fit.<br>- EFA yielded a 23-item five-factor model corresponding to Fear of Fat (item 1, 2, 10, 11, 12, 14), Diet (item 6, 16, 17), Other's Opinions (item 8, 13, 20), Preoccupation with Food (item 3, 4, 18, 21), Food Enjoyment (item 15). Items 5, 22, 26 were discarded
Rutherford et al. [28]	EAT-26	United Kingdom/English	Twins	580	- EFA supported a unidimensional model. Items 5, 8, 9, 13, 15, 19, 25, 26 were discarded.
Spivak-Lavi et al. [29]	EAT-26	Israel/Hebrew & Arabic	Community sample	2,614	- CFA supported Garner et al. [24] 26-item three-factor model in Israeli populations.<br>- EFA yielded a two-factor model in Israeli Jews with 23 items; Binge-purge and restricting (items 2, 4, 5, 7, 8, 9, 10, 15, 16, 20 24, 26), Dieting and preoccupation with food/weight/shape (items 1, 3, 6, 11, 12, 14, 17, 18, 21, 22, 23). A four-factor model in Israeli Arab Muslims with 19 items; Binge-purge/restricting /dieting (items 2, 7, 9, 16, 17, 23, 24, 26), Preoccupation with food/weight/shape (item 1, 11, 12, 14), Fear of loss of control over eating (item 3, 4, 18, 21), Eating related to social pressure (item 8, 13, 20). A two-factor model in Israeli Arab Christians with 20 items; Binge-purge/dieting (items 2, 4, 7, 8, 9, 10, 16, 17, 18, 20, 21, 22, 23, 24, 26), Preoccupation with food/weight/shape (items 1, 11, 12, 14, 19).
Szabo and Allwood [30]	EAT-26	South Africa/ Zulu	Female university students	361	- EFA supported a 26-item three-factor model.

Note. CFA = confirmatory factor analysis, EFA = exploratory factor analysis, ED = eating disorder. EAT-40 and Children's EAT (ChEAT) versions excluded from literature review.

References

1. Ahmadi, S.; R. Moloodi; M.-R. Zarbaksh; A. Ghaderi. Psychometric properties of the Eating Attitude Test-26 for female Iranian students. *Eat Weight Disord* **2014**, *19*, 183-189, doi: 10.1007/s40519-014-0106-7
2. Ambrosi-Randić, N.; A. Pokrajac-Bulian. Psychometric properties of the eating attitudes test and children's eating attitudes test in Croatia. *Eat Weight Disord* **2005**, *10*, e76-e82, doi: 10.1007/BF03327495
3. Belon, K.E.; J.E. Smith; A.D. Bryan; D.N. Lash; J.L. Winn; L.M. Gianini. Measurement invariance of the Eating Attitudes Test-26 in Caucasian and Hispanic women. *Eat Behav* **2011**, *12*, 317-320, doi: 10.1016/j.eatbeh.2011.07.007
4. Rutt, C.D.; K.J. Coleman. The Evaluation of a Measurement Model for the Body Image Questionnaire and the Eating Attitudes Test in a Hispanic Population. *Hisp J Behav Sci* **2001**, *23*, 153-170, doi: 10.1177/0739986301232003
5. Ocker, L.B.; E.T.C. Lam; B.E. Jensen; J.J. Zhang. Psychometric Properties of the Eating Attitudes Test. *Meas Phys Educ Exerc Sci* **2007**, *11*, 25-48, doi: 10.1080/10913670709337010
6. Choudry, I.Y.; D.B. Mumford. A pilot study of eating disorders in Mirpur (Pakistan) using an Urdu version of the eating attitudes test. *Int. J. Eat. Disord* **1992**, *11*, 243-251, doi: 10.1002/1098-108X(199204)11:3<243::AID-EAT2260110307>3.0.CO
2-P
7. Doninger, G.L.; C.K. Enders; K.F. Burnett. Validity Evidence for Eating Attitudes Test Scores in a Sample of Female College Athletes. *Meas Phys Educ Exerc Sci* **2005**, *9*, 35-49, doi: 10.1207/s15327841mpee0901_3
8. Koslowsky, M.; Z. Scheinberg; A. Bleich; M. Mark; A. Apter; Y. Danon; Z. Solomon. The Factor Structure and Criterion Validity of the Short Form of the Eating Attitudes Test. *J Pers Assess* **1992**, *58*, 27-35, doi: 10.1207/s15327752jpa5801_3
9. Dotti, A.; R. Lazzari. Validation and reliability of the Italian EAT-26. *Eat Weight Disord* **1998**, *3*, 188-194, doi: 10.1007/BF03340009
10. Douka, A.; E. Grammatopoulou; E. Skordilis; D. Koutsouki. Factor analysis and cut-off score of the 26-item eating attitudes test in a Greek sample. *Biology of exercise* **2009**, *5*, doi: 10.4127/jbe.2009.0025
11. Engelsen, B.K.; K.A. Hagtvet. The dimensionality of the 12-item version of the Eating Attitudes Test. Confirmatory factor analyses. *Scand J Psychol* **1999**, *40*, 293-300, doi: 10.1111/1467-9450.404129
12. Fekih-Romdhane, F.; S. Obeid; D. Malaeb; R. Hallit; S. Hallit. Validation of a shortened version of the Eating Attitude Test in the Arabic language. *Journal of eating disorders* **2022**, *10*, 1-127, doi: 10.1186/s40337-022-00651-5
13. Haddad, C.; C. Khoury; P. Salameh; H. Sacre; R. Hallit; N. Kheir; S. Obeid; S. Hallit. Validation of the Arabic version of the Eating Attitude Test in Lebanon: a population study. *Public Health Nutr* **2021**, *24*, 4132-4143, doi: 10.1017/S1368980020002955
14. Johnson, C.S.; J. Bedford. Eating attitudes across age and gender groups: a Canadian study. *Eat Weight Disord* **2004**, *9*, 16-23, doi: 10.1007/BF03325040
15. Kelly, N.R.; K.S. Mitchell; R.W. Gow; S.E. Trace; J.A. Lydecker; C.E. Bair; S. Mazzeo. An Evaluation of the Reliability and Construct Validity of Eating Disorder Measures in White and Black Women. *Psychol Assess* **2012**, *24*, 608-617, doi: 10.1037/a0026457
16. Khaled, S.M.; L. Kimmel; K. Le Trung. Assessing the factor structure and measurement invariance of the eating attitude test (EAT-26) across language and BMI in young Arab women. *J Eat Disord* **2018**, *6*, 14-14, doi: 10.1186/s40337-018-0199-x

17. King, M.B.; D. Bhugra. Eating disorders: lessons from a cross-cultural study. *Psychol. Med* **1989**, *19*, 955-958, doi: 10.1017/S0033291700005675
18. Lavik, N.J.; S.E. Clausen; W. Pedersen. Eating behaviour, drug use, psychopathology and parental bonding in adolescents in Norway. *Acta Psychiatr Scand* **1991**, *84*, 387-390, doi: 10.1111/j.1600-0447.1991.tb03164.x
19. Lee, S.; A.M. Lee. Disordered eating in three communities of China: A comparative study of female high school students in Hong Kong, Shenzhen, and rural Hunan. *Int. J. Eat. Disord* **2000**, *27*, 317-327, doi: 10.1002/(SICI)1098-108X(200004)27:3<317::AID-EAT9>3.0.CO

2-2
20. Maïano, C.; A.J.S. Morin; M.-C. Lanfranchi; P. Therme. The Eating Attitudes Test-26 Revisited using Exploratory Structural Equation Modeling. *J Abnorm Child Psychol* **2013**, *41*, 775-788, doi: 10.1007/s10802-013-9718-z
21. McEnery, F.; A. Fitzgerald; F. McNicholas; B. Dooley. Fit for Purpose, Psychometric Assessment of the Eating Attitudes Test-26 in an Irish Adolescent Sample. *Eat Behav* **2016**, *23*, 52-57, doi: 10.1016/j.eatbeh.2016.07.006
22. Mumford, D.B.; A.M. Whitehouse; I.Y. Choudry. Survey of eating disorders in English-medium schools in Lahore, Pakistan. *Int. J. Eat. Disord* **1992**, *11*, 173-184, doi: 10.1002/1098-108X(199203)11:2<173::AID-EAT2260110208>3.0.CO

2-L
23. Mumford, D.B.; A.M. Whitehouse; M. Platts. Sociocultural Correlates of Eating Disorders among Asian Schoolgirls in Bradford. *Br J Psychiatry* **1991**, *158*, 222-228, doi: 10.1192/bjp.158.2.222
24. Garner, D.M.; M.P. Olmsted; Y. Bohr; P.E. Garfinkel. The Eating Attitudes Test: Psychometric features and clinical correlates. *Psychol Med* **1982**, *12*, 871-878, doi: 10.1017/s0033291700049163
25. Park, J.; M.P. Beaudet. Eating attitudes and their correlates among Canadian women concerned about their weight. *Eur. Eat. Disorders Rev* **2007**, *15*, 311-320, doi: 10.1002/erv.741
26. Rivas, T.; R. Bersabé; M. Jiménez; C. Berrocal. The Eating Attitudes Test (EAT-26): Reliability and Validity in Spanish Female Samples. *Span J Psychol* **2010**, *13*, 1044-1056, doi: 10.1017/S1138741600002687
27. Rogoza, R.; A. Brytek-Matera; D.M. Garner. Analysis of the EAT-26 in a non-clinical sample. *Arch. Psychiatry Psychother* **2016**, *18*, 54-58, doi: 10.12740/APP/63647
28. Rutherford, J.; P. McGuffin; R.J. Katz; R.M. Murray. Genetic influences on eating attitudes in a normal female twin population. *Psychol. Med* **1993**, *23*, 425-436, doi: 10.1017/S003329170002852X
29. Spivak-Lavi, Z.; O. Peleg; O. Tzischinsky; D. Stein; Y. Latzer. Differences in the Factor Structure of the Eating Attitude Test-26 (EAT-26) in Different Cultures in Israel: Jews, Muslims, and Christians. *Nutrients* **2021**, *13*, 1899, doi: 10.3390/nu13061899
30. Szabo, C.P.; C.W. Allwood. Application of the Eating Attitudes Test (EAT-26) in a rural, Zulu speaking, adolescent population in South Africa. *World Psychiatry* **2004**, *3*, 169-71, doi:
